# The SNX-PX-BAR Family in Macropinocytosis: The Regulation of Macropinosome Formation by SNX-PX-BAR Proteins

**DOI:** 10.1371/journal.pone.0013763

**Published:** 2010-10-29

**Authors:** Jack T. H. Wang, Markus C. Kerr, Seetha Karunaratne, Angela Jeanes, Alpha S. Yap, Rohan D. Teasdale

**Affiliations:** Institute for Molecular Bioscience and Australia Research Council (ARC) Centre of Excellence in Bioinformatics, The University of Queensland, St. Lucia, Brisbane, Australia; University of Nebraska Medical Center, United States of America

## Abstract

**Background:**

Macropinocytosis is an actin-driven endocytic process, whereby membrane ruffles fold back onto the plasma membrane to form large (>0.2 µm in diameter) endocytic organelles called macropinosomes. Relative to other endocytic pathways, little is known about the molecular mechanisms involved in macropinocytosis. Recently, members of the Sorting Nexin (SNX) family have been localized to the cell surface and early macropinosomes, and implicated in macropinosome formation. SNX-PX-BAR proteins form a subset of the SNX family and their lipid-binding (PX) and membrane-curvature sensing (BAR) domain architecture further implicates their functional involvement in macropinosome formation.

**Methodology/Principal Findings:**

We exploited the tractability of macropinosomes through image-based screening and systematic overexpression of SNX-PX-BAR proteins to quantitate their effect on macropinosome formation. SNX1 (40.9+/−3.19 macropinosomes), SNX5 (36.99+/−4.48 macropinosomes), SNX9 (37.55+/−2.4 macropinosomes), SNX18 (88.2+/−8 macropinosomes), SNX33 (65.25+/−6.95 macropinosomes) all exhibited statistically significant (p<0.05) increases in average macropinosome numbers per 100 transfected cells as compared to control cells (24.44+/−1.81 macropinosomes). SNX1, SNX5, SNX9, and SNX18 were also found to associate with early-stage macropinosomes within 5 minutes following organelle formation. The modulation of intracellular PI(3,4,5)P_3_ levels through overexpression of PTEN or a lipid phosphatase-deficient mutant PTEN(G129E) was also observed to significantly reduce or elevate macropinosome formation respectively; coexpression of PTEN(G129E) with SNX9 or SNX18 synergistically elevated macropinosome formation to 119.4+/−7.13 and 91.4+/−6.37 macropinosomes respectively (p<0.05).

**Conclusions/Significance:**

SNX1, SNX5, SNX9, SNX18, and SNX33 were all found to elevate macropinosome formation and (with the exception of SNX33) associate with early-stage macropinosomes. Moreover the effects of SNX9 and SNX18 overexpression in elevating macropinocytosis is likely to be synergistic with the increase in PI(3,4,5)P_3_ levels, which is known to accumulate on the cell surface and early-stage macropinocytic cups. Together these findings represent the first systematic functional study into the impact of the SNX-PX-BAR family on macropinocytosis.

## Introduction

Macropinocytosis is a high-capacity variant of endocytic uptake first reported by Warren Lewis in 1931 [1], generating large endocytic organelles termed macropinosomes through actin-driven evaginations of the plasma membrane. Unlike clathrin-mediated endocytosis or phagocytosis, macropinocytosis is not regulated by the binding of cargo to the receptors which then recruit effector molecules that aid in vesicle formation [2]; instead the activation of receptor tyrosine-kinases (RTK) in response to growth factor treatment drives the actin-mediated ruffling of the plasma membrane, non-selectively engulfing large volumes of fluid to form phase bright macropinosomes larger than 0.2 µm in diameter [1], [3]. Strikingly, this heterogeneous size range is significantly larger than other endocytic compartments such as clathrin-coated vesicles (85−110 nm), caveolae (55−75 nm), and clathrin-independent carrier/Glycosylphosphatidylinositol (GPI)-anchored protein-enriched early endosomal compartments (CLIC/GEEC) (40−80 nm), which together with its mechanism of formation distinguishes macropinocytosis from other endocytic pathways [4], [5], [6], [7], [8].

The rapid and large fluid-carrying capacity of macropinocytosis is central to its many diverse physiological roles. Within the immune response, macropinocytosis is particularly active within antigen-presenting cells before presenting the antigenic peptides on the cell surface [9]. Cells overexpressing oncogenes have also been shown to exhibit elevated levels of macropinocytosis [10], [11], [12], and treatment with growth factors associated with uncontrolled cell proliferation in cancerous tissue transiently upregulates this pathway [13], [14], [15], [16]. Moreover due to its non-specific and high capacity nature of fluid intake, macropinocytosis is an ideal route for pathogens to hijack in order to gain entry into the cell [17]. The invasion of *Salmonella enterica* Serovar typhimurium [18], [19], *Shigella flexneri* [20], [21], [22], *Mycobacterium* [23], *Vaccinia virus* [24], and *Coxsackievirus* [25] have all been connected to the exploitation of macropinocytosis.

Despite its significant physiological implications, there is a paucity of knowledge regarding macropinocytosis relative to other endocytic pathways; this can be largely attributed to the inability to definitively characterize macropinosomes through a stable association between the organelle and specific proteins or lipids. It is known however that treatment with millimolar concentrations of the ion exchange inhibitor amiloride inhibits macropinocytosis but not clathrin-mediated endocytosis [26]. Amiloride has been associated with the lowering of submembranous pH and preventing Rho GTPase signalling and actin remodeling [27], both of which are essential for the membrane ruffling necessary for macropinocytosis. This property can be used to define macropinocytosis along with the size of the organelle and responsiveness to growth factor stimulation [3].

PI(3)K activity has also been shown to be required for macropinosome formation [10], [28], implicating the direct role of its phosphorylation targets, 3-phosphoinositides, in the process. Phosphoinositides (PI) result from the phosphorylation of phosphatidylinositol at different positions along the inositol ring [29], and different phosphoinositide species are crucial for the formation and maturation of macropinosomes. PI(4,5)P_2_ levels on membrane ruffles have been observed to be more than double the amount present on planar membranes, rapidly dropping just prior to macropinosome closure [30]. Conversely, PI(3,4,5)P_3_ levels increase locally at the site of macropinosome formation and peak when the macropinosome closes [30], [31] and the subsequent drop in PI(3,4,5)P_3_ levels coincides with the accumulation of PI(3)P on the body of the macropinosome [32]. Rab5 and its effectors have been implicated in this stage of the macropinosome lifecycle, as Rab5 recruitment to the macropinosome occurs prior to PI(3,4,5)P_3_ loss [31], and it is known to interact with Vps34-p150 to synthesize PI(3)P [33]. Moreover the Rab5 effector Rabankyrin-5 binds to PI(3)P by virtue of its FYVE domain, and is directly involved in regulating macropinosome formation; the overexpression and siRNA-mediated depletion of Rabankyrin-5 increases and decreases the number of macropinosomes formed, respectively [34].

Given the precise spatiotemporal regulation of the phosphoinositide composition on the macropinosome body at different stages in its lifecycle, proteins that bind to and/or regulate the synthesis of phosphoinositides would potentially be involved in macropinocytosis. The Phox homology (PX) domain is a 100−140 residue phosphoinositide-binding domain that has been found in 15 yeast proteins and 47 mammalian proteins [35]. Its most well-established function is targeting proteins to phosphoinositide-rich membranes; all 15 PX-domain proteins in yeast have been shown to interact specifically with PI(3)P [36] and many of the mammalian PX-domain proteins bind to a wide variety of phosphoinositide species [37], [38], [39], [40], [41],[42],[43],[44],[45],[46]. Of the 47 mammalian PX-domain proteins, 30 are within the Sorting Nexin family [35]. A subset of the Sorting Nexin family also contains a C-terminal Bin/Amphiphysin/Rvs (BAR) domain, proposed to be involved in protein dimerization, sensing membrane curvature, and membrane tubulation [47], [48], [49], [50]. It was further demonstrated that as a mechanism of protein targeting and recruitment, the PX and BAR domains cooperate in the coincidence detection of highly-curved phosphoinositide-rich membranes [43], [51]. 12 out of the 30 Sorting Nexins contain both PX and BAR domains - SNX1, SNX2, SNX4, SNX5, SNX6, SNX7, SNX8, SNX9, SNX18, SNX30, SNX32 and SNX33; collectively these proteins form the SNX-PX-BAR family. Three of the SNX-PX-BAR proteins also possess an additional N-terminal SH3 domain – SNX9, SNX18 and SNX33. Together, these three proteins form the SH3-PX-BAR subgroup.

We have previously demonstrated that SNX5 is involved early in the macropinocytic lifecycle, regulating the formation of macropinosomes at the cell surface [14], [52]. SNX5 is transiently recruited to the plasma membrane in response to EGF [45], a physiological treatment known to upregulate macropinocytosis [13]. This is likely due to the elevation in PI(3,4)P_2_ on the plasma membrane following EGF treatment, to reflect the PI(3,4)P_2_-specificity of the PX domain of SNX5 [45]. Following its cell-surface translocation, SNX5 can be localized to discrete subdomains of the macropinosome along with Rab5 and Rabankyrin-5 early in the macropinosome lifecycle, forming extensive microtubule-dependent tubules that depart from the macropinosome body [14]. This extensive tubulation removes a significant portion of the limiting membrane of the macropinosome, and is a potential mechanism for rapid membrane and protein trafficking back to the cell surface to facilitate further macropinocytic events; this model is consistent with our observation that the stable overexpression of SNX5 led to a 2-fold elevation in macropinosome formation [52].

Within this study, we aimed to extend our understanding of the molecular coordination involved in regulating macropinocytosis by discovering novel molecules that influence macropinosome formation. We hypothesize that proteins that share the PX and BAR domain architecture of SNX5 – the SNX-PX-BAR family - may also be involved in macropinocytosis. The twelve members of the SNX-PX-BAR family were systematically assayed using a quantitative image-based assay for macropinosome formation, and it was observed that five candidates within the family - SNX1, SNX5, SNX9, SNX18 and SNX33 - all elevated macropinosome formation. Of these five candidates, only SNX1, SNX5, SNX9, and SNX18 could be found to associate with early-stage macropinosomes five minutes post formation, whereas SNX33 was observed to be cytosolic in its subcellular distribution. The connection between the phosphoinositide-binding capacity of the PX-BAR domain and its ability to influence macropinosome formation was further investigated by modulating the levels of PI(3,4,5)P_3_, which has been localized to the macropinocytic cup very early in the formation process [31]. Overexpression of Phosphatase and tensin homolog (PTEN), the metabolic enzyme which catalyzes the dephosphorylation of PI(3,4,5)P_3_ to PI(4,5)P_2_, or a lipid phosphatase-deficient mutant PTEN(G129E), significantly decreased or increased macropinosome formation respectively. Moreover coexpressing PTEN(G129E) with either SNX9 or SNX18, both reported to preferentially bind PI(4,5)P_2_ through their respective PX-BAR domains [46], resulted in a synergistic elevation in macropinosome formation. These results suggest a mechanistic link may exist between the conversion from PI(4,5)P_2_ to PI(3,4,5)P_3_ and SNX-PX-BAR proteins that are able to selectively bind to these phosphoinositides in the regulation of macropinosome formation.

The results of this screen represent the first functional study of the SNX-PX-BAR family in macropinocytosis, as well as implicating the role of phosphoinositide levels in macropinosome formation.

## Results

### Screening Assay Development and Validation

**Figure 1:**
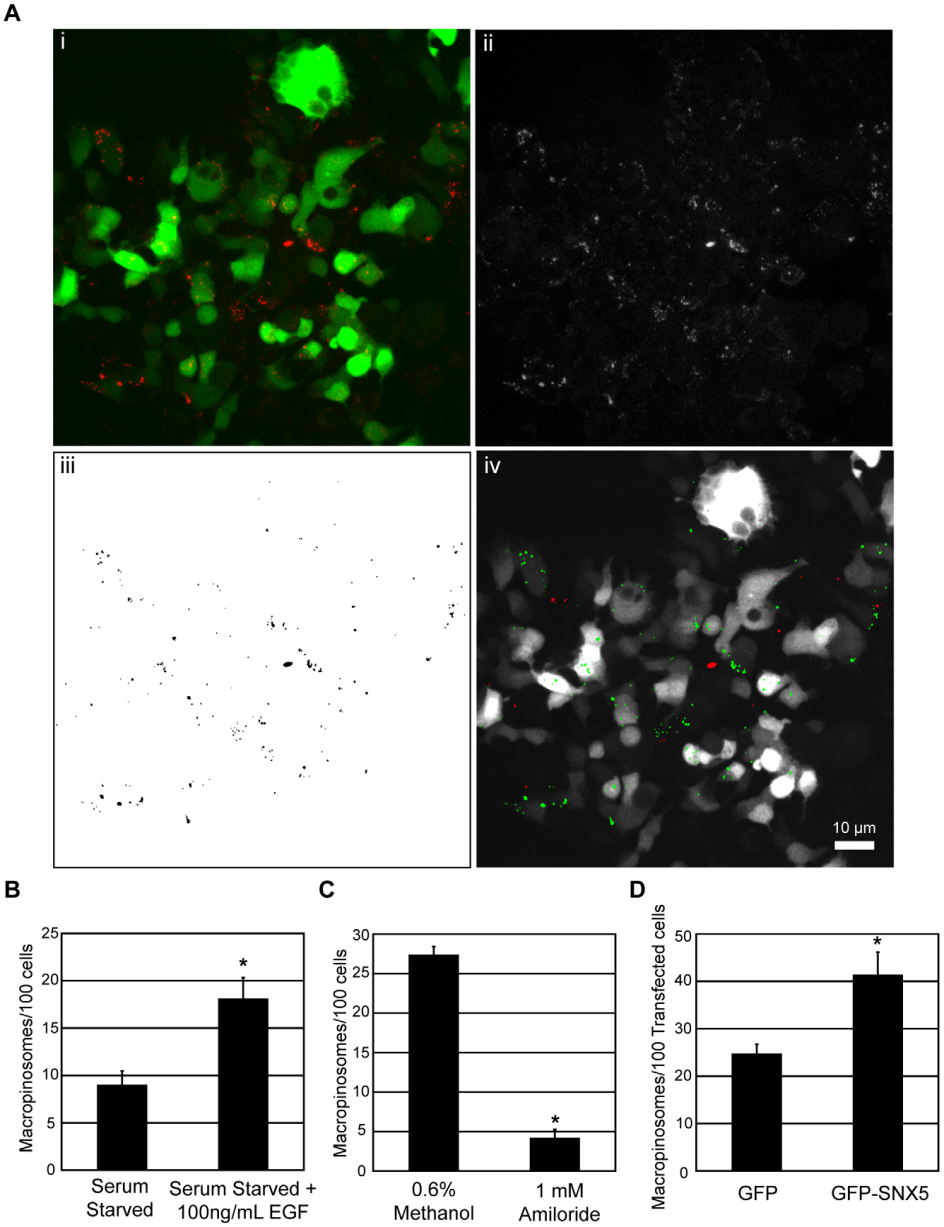
Macropinosome formation screening assay validation. A: 24 hours post transfection, HEK-Flp-In cell monolayers were pulsed for 5 minutes with 100 µg/mL dextran (10,000 MW) conjugated to tetramethylrhodamine (dextran-TR) at 37°C. The samples were then washed in 4°C PBS, fixed in 4% PFA and imaged and processed as described in Materials and Methods. Briefly Z-stack images comprising of 3×5 µm Z slices were merged into a single RGB image of transfected cells (green) stained with dextran-TR (red) (Ai). The red channel from the RGB image was isolated and converted to an 8-bit grayscale image (Aii). Dextran-positive macropinosomes were selected based on size (>0.5 µm in diameter) and fluorescent intensity (>100). Selected macropinosomes are shown in the foreground as black (Aiii). This binary image was then converted to a mask and superimposed onto the Green channel of the original RGB image to measure the green fluorescent intensity of the area occupied by each macropinosome in the image (Aiv). The particles with green fluorescence intensity higher than background signal (>20) were considered to be macropinosomes within a transfected cell, represented in green. Red particles represent discarded macropinosomes determined to be outside of a transfected cell. Scale  = 10 µm. B, C, D: HEK-Flp-In cell monolayers were either serum-starved for 16 hours and treated with dextran-TR in the presence or absence of 100 ng/mL EGF for 5 minutes at 37°C (B), treated with 1 mM amiloride or carrier (0.6% Methanol) for 30 minutes at 37°C before pulsing with dextran-TR (C), or transiently transfected with pEGFP-C1 or pEGFP-SNX5 before pulsing with dextran-TR (D). The samples in each case were assayed for macropinosome formation as described in Materials and Methods, quantitating the mean number of macropinosomes/100 transfected cells over 3 replicates of 500 transfected cells for each condition. * denotes statistical significance (p<0.05) using the Student's T-test. Error bars denote Standard Error of the Mean (S.E.M).

To test the hypothesis that the SNX-PX-BAR family is involved in macropinocytosis, a functional assay was developed in order to screen the candidate proteins in a systematic fashion. We chose to exploit the visual tractability of macropinosomes via light microscopy to develop an image-based quantitative assay for macropinosome formation. Cells were transfected with the protein of interest and treated to specific cellular conditions before being pulsed for 5 minutes with fluorescently-conjugated dextran (10,000 MW). This time point was selected as macropinosomes formed in this period show limited maturation [14], [52]. The samples were then processed for confocal imaging and quantitated through an automated image analysis protocol (Figure 1A). Briefly, for each field of view the channels corresponding to GFP and dextran fluorescence were captured (Figure 1Ai). The total number of dextran-positive macropinosomes within the field of view was then quantified by size (>0.5 µm in diameter) and fluorescent intensity (>100) (Figure 1Aii and Aiii). Each macropinosome was then used as a mask to create a region of interest within the image, and correlated to the same position within the green channel (Figure 1Aiv). This allowed the measurement of the green fluorescent intensity at the position of each macropinosome within the image, and any intensity higher than background signal (>20) was indicative of presence within a GFP-positive transfected cell. Other macropinosomes that correlated to green intensities below this threshold were discarded. The green channel also allowed for the calculation of the number of transfected cells within the field of view, and the mean number of macropinosomes per 100 transfected cells was measured for each overexpressed construct.

To validate the specificity and sensitivity of this methodology, we applied the screening assay to cellular conditions and proteins of interest known to regulate macropinocytosis. EGF has been shown to rapidly stimulate macropinocytosis [13], and using the assay developed, a 2 fold increase in macropinosome formation (p<0.05) was observed in serum-starved cells treated with 100 ng/mL EGF for 5 minutes relative to control samples (Figure 1B). Treatment with 1 mM Amiloride, a disruptor of Na+/H+ exchange known to specifically inhibit macropinocytosis [26], resulted in a 4.54 fold drop in macropinosome number (p<0.05) relative to cells treated with carrier (0.6% Methanol), indicating that the structures visualized through the assay are amiloride-sensitive (Figure 1C). Moreover upon overexpression of pEGFP-SNX5, which has been reported to be localized on early-stage macropinosomes [14] as well as regulating the rate at which macropinosomes are formed [52], a 1.5 fold increase in macropinosome formation (p<0.05) was observed compared to cells expressing pEGFP-C1 (Figure 1D). Together these results suggest that the structures as detected by the screening assay are transiently upregulated by EGF treatment, and sensitive to amiloride-mediated inhibition of macropinocytosis, both of which are hallmarks specific to macropinocytosis [3]. Furthermore the screening assay is sufficiently sensitive to detect an elevation in macropinosome formation upon overexpression of SNX5, a known regulator of macropinosome formation [52].

**Figure 2:**
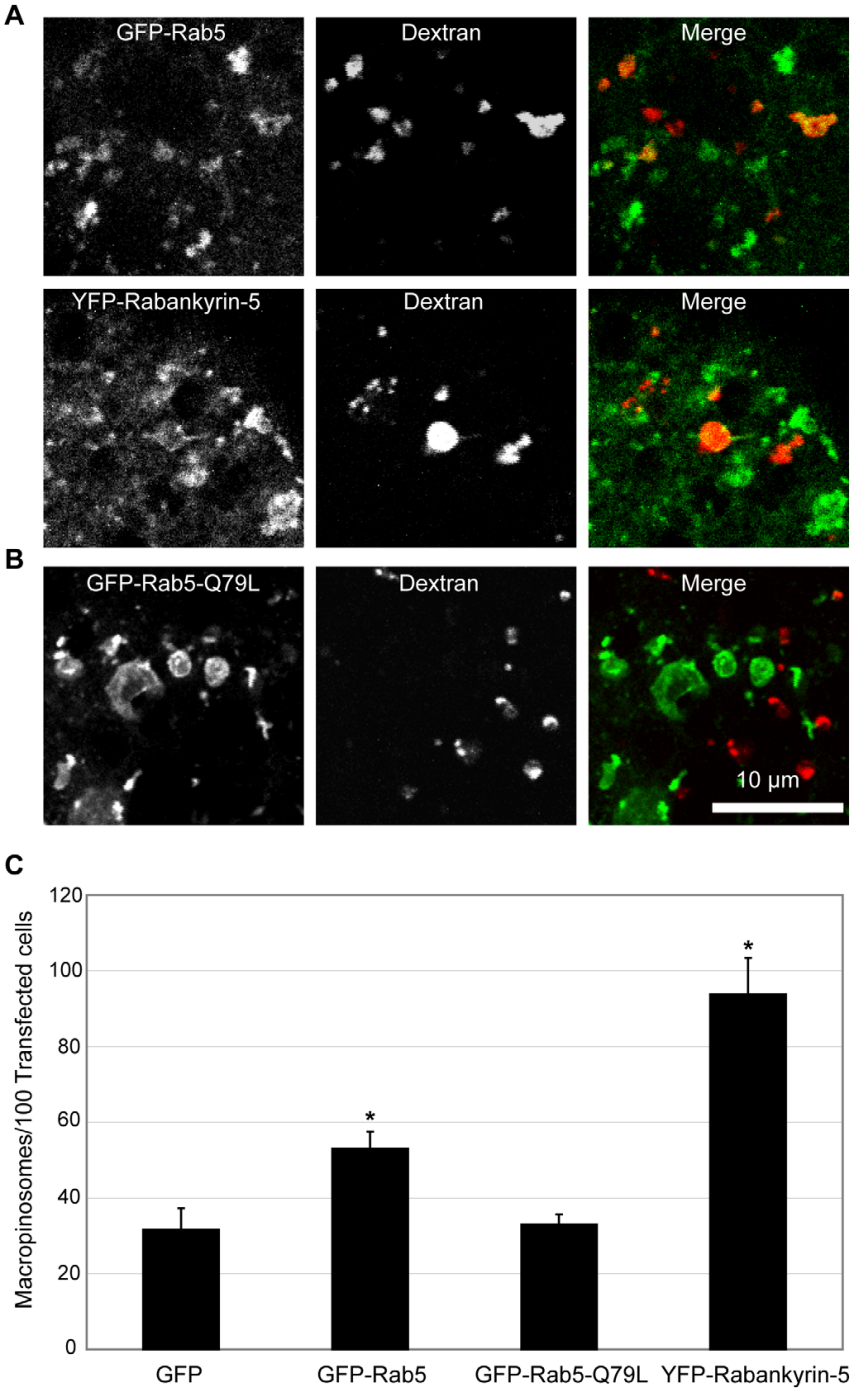
Rab5 and Rabankyrin5 overexpression increases macropinosome formation. The distribution of Rab5, Rabankyrin-5 (A) or Rab5(Q79L) (B) in green relative to dextran-positive macropinosomes (red) Scale bar  = 10 µm. (C) HEK-Flp-In cells were transiently transfected with pEGFP-C1, pEGFP-Rab5, pEGFP-Rab5-Q79L and pEYFP-Rabankyrin-5. The mean number of macropinosomes/100 transfected cells was quantitated over 3 replicates of 500 transfected cells for each condition. * denotes statistical significance (p<0.05) using the Student's T-test, performing pairwise analyses relative to cells transfected with pEGFP-C1 alone. Error bars denote S.E.M.

Rab5 and its downstream effector Rabankyrin 5 are also regulators of macropinocytosis [34] and were overexpressed in HEK-Flp-In cells and pulsed with dextran for 5 minutes. Large macropinosomes could be seen at this early timepoint which are positive for GFP-Rab5, and the same was observed for YFP-Rabankyrin-5 (Figure 2A), consistent with their localization in EGF-stimulated A431 cells [34]. Moreover the specificity of the assay was contingent on the significantly larger size of macropinosomes (0.2 to 5 µm in diameter) relative to other endocytic organelles (up to 0.1 µm in diameter), but it was formally possible for the promotion of homotypic fusion to increase the size of smaller endosomes. To test this, the constitutively active Rab5-Q79L mutant known to promote endosomal homotypic fusion [53], was overexpressed in HEK-Flp-In cells. Enlarged endosomes were observed in pEGFP-Rab5-Q79L-expressing cells within the diameter range of macropinosomes, but after 5 minutes of dextran uptake, none of these structures were dextran positive (Figure 2B). This indicates that under these conditions, the enlarged endosomes were the result of homotypic fusion between intracellular early endosomes, which can be specifically distinguished from large dextran-positive macropinosomes derived from the cell surface following 5 minutes of uptake.

Utilising the screening assay, a 1.7 fold elevation in macropinocytosis (p<0.05) was observed in pEGFP-Rab5 overexpressing cells relative to pEGFP-C1-overexpressing cells. Moreover a 3 fold elevation in macropinosome formation (p<0.05) was observed for cells overexpressing pEYFP-Rabankyrin-5 (Figure 2C). The difference in the degree of macropinocytosis elevation was consistent with Rabankyrin-5 being a downstream effector of Rab5 that is directly involved in macropinocytosis [34]; elevating Rab5 levels would promote the activation of an existing endogenous pool of Rabankyrin-5, whereas elevating Rabankyrin-5 would have a direct and more pronounced effect on macropinocytosis. No difference was observed in macropinocytosis between cells expressing pEGFP-C1 or pEGFP-Rab5-Q79L, further indicating that the enlarged endosomes are not detected as false positives within the assay.

### SNX1, SNX5, SNX9, SNX18 and SNX33 are involved in macropinosome formation

**Figure 3:**
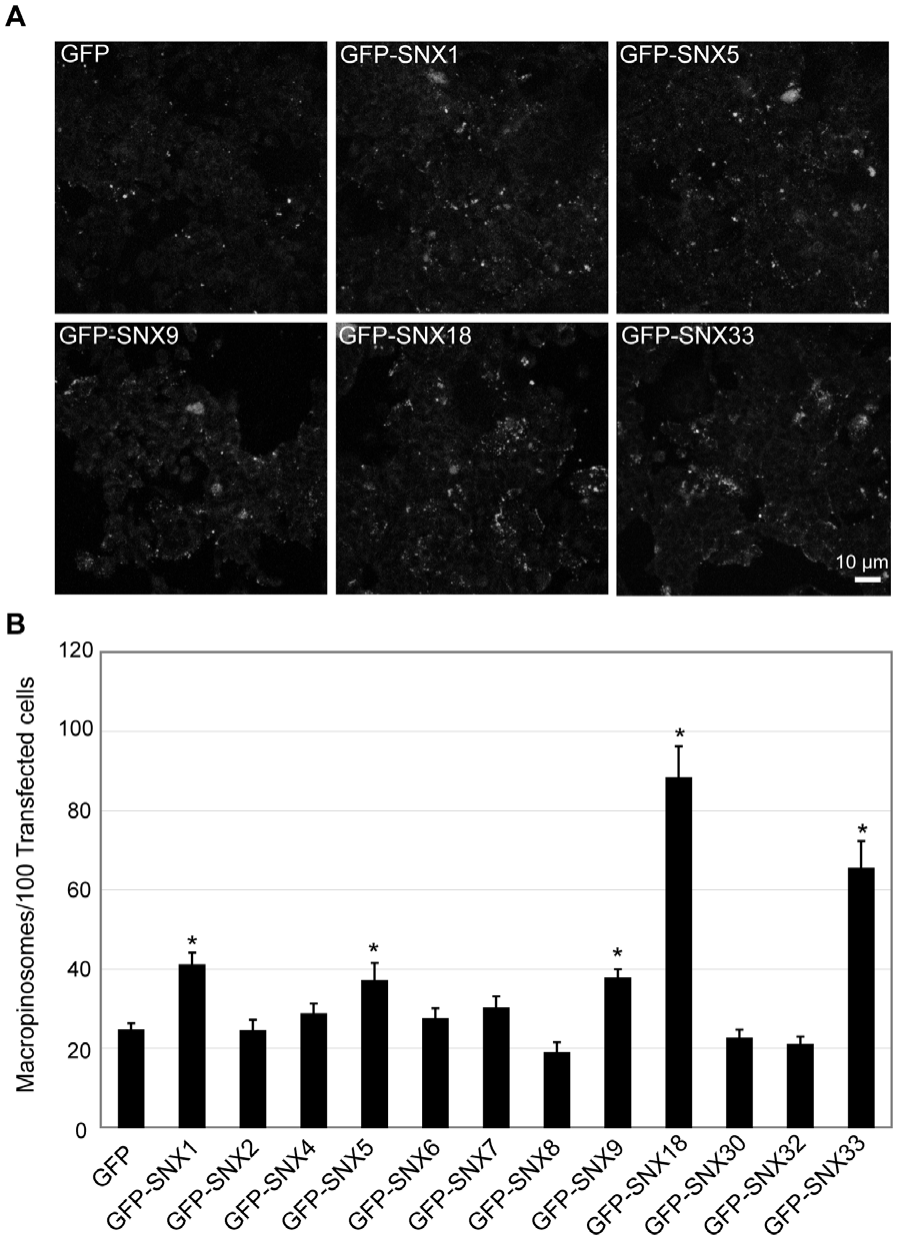
The SNX-PX-BAR family is involved in macropinosome formation. HEK-Flp-In cells transiently overexpressing GFP-tagged members of the SNX-PX-BAR family were assayed for macropinosome formation as described in Materials and Methods. A: Dextran-TR labeling of cells transfected with the specified constructs. Scale bar  = 10 µm. B: The mean number of macropinosomes/100 transfected cells was quantitated over 8 replicates of 500 transfected cells for each condition. * denotes statistical significance (p<0.05) using the Student's T-test, performing pairwise analyses relative to cells transfected with pEGFP-C1 alone. Error bars denote S.E.M.

Following the validation of the screening assay using the known regulators of macropinocytosis, the gain-of-function macropinosome formation screen was conducted on each member of the SNX-PX-BAR family. 24 hours following the transfection, cells were assayed for macropinosome formation across 8 replicates of at least 500 transfected cells per construct in order to obtain the average macropinosomes formed/100 transfected cell. Relative to the macropinosomes formed in cells overexpressing pEGFP-C1, pEGFP-SNX1, pEGFP-SNX5 and pEGFP-SNX9 overexpressing cells all exhibited a 2 fold elevation in macropinocytosis (Figure 3A and B). SNX5 [14], [52] and SNX9 [40] have both been implicated in macropinosome formation, so they serve as positive controls for the sensitivity of the assay. The promotion of macropinosome formation following SNX1 overexpression is also consistent with its interaction with SNX5 [14], and presumably the two proteins regulate macropinocytosis through a common mechanism. The overexpression of pEGFP-SNX18 and pEGFP-SNX33 also resulted in 3.6 and 2.7 fold elevations in macropinocytosis respectively (Figure 3). Therefore the increased levels of SNX1, SNX5, SNX9, SNX18 and SNX33 all significantly elevated the number of macropinosomes formed. with the SH3-PX-BAR subgroup showing the highest elevations. Quantitative fluorescence analysis captured under identical non-saturating conditions revealed no significant difference between total GFP fluorescence across cell monolayers overexpressing pEGFP-SNX9, pEGFP-SNX18, or pEGFP-SNX33.

**Figure 4:**
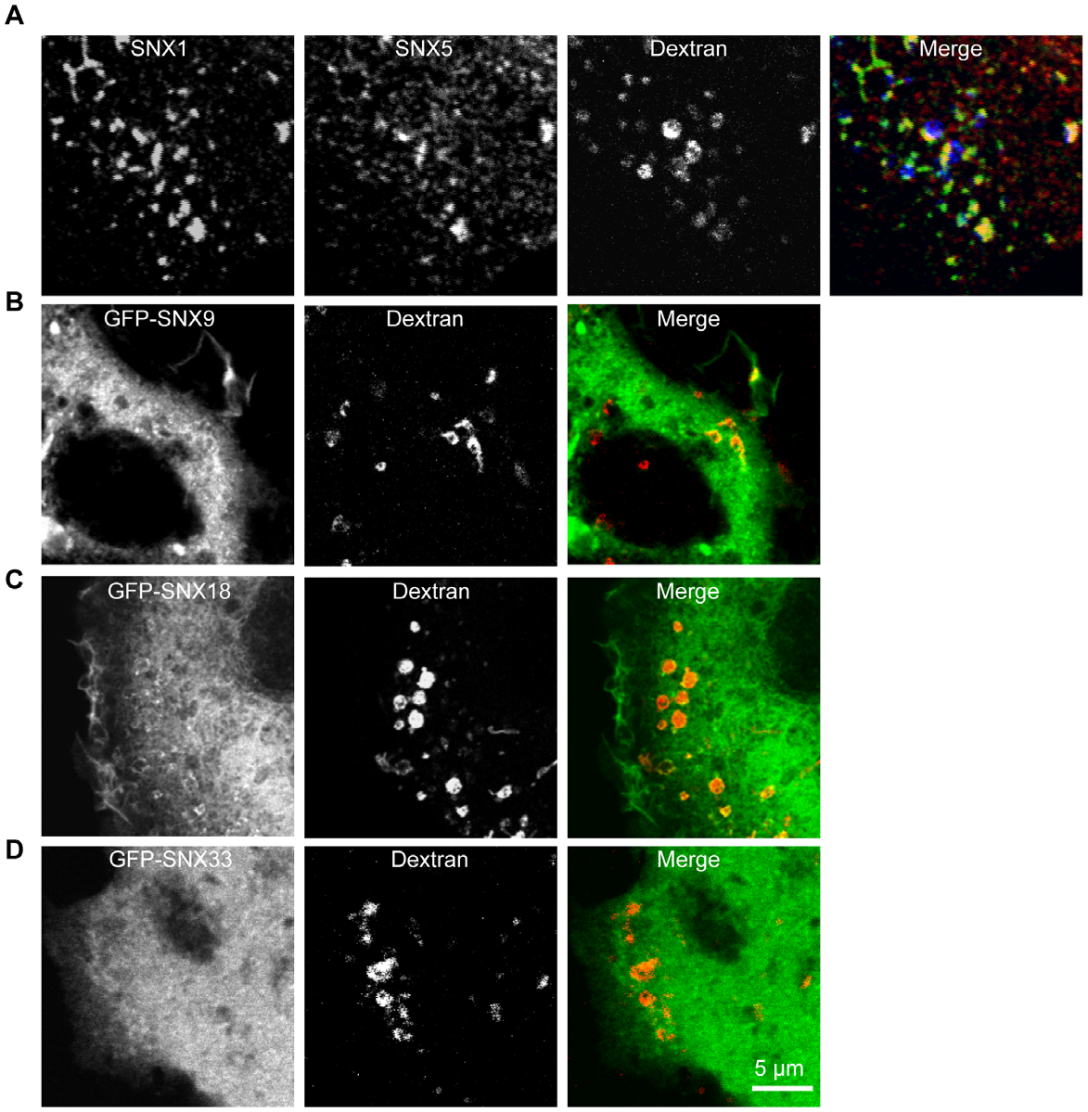
SNX1, SNX5, SNX9 and SNX18 associate with early macropinosomes. A: HEK-Flp-In cells were pulsed with 100 µg/mL dextran (10,000 MW) conjugated to Alexa-647 (dextran-647) for 5 minutes before being transferred to 4°C and washed with 0.45 mM CaCl_2_ 1 mM MgCl_2_ PBS. The cells were then treated with 80 U/mL Streptolysin O for 5 minutes at 4°C to only permeabilize the plasma membrane before washing in 0.45 mM CaCl_2_ 1 mM MgCl_2_ PBS and incubating with 37°C PBS for 5 minutes. Following fixation with 4% PFA for 30 minutes at 4°C, cell monolayers are incubated with monoclonal and polyclonal antibodies against SNX1 and SNX5 respectively, followed by Alexa-488-conjugated goat-anti-mouse and Cy3-conjugated goat-anti-rabbit IgG secondary antibodies. B, C, and D: 24 hours post transfection, HEK-Flp-In cells transfected with pEGFP-SNX9 (B), pEGFP-SNX18 (C) and pEGFP-SNX33 (D) were pulsed with dextran-TR for 5 minutes at 37°C prior to fixation at 4°C in 4% PFA. Images were collected on an LSM 510 Meta confocal microscope. Scale bar  = 5 µm.

The question posed next was whether or not these SNX-PX-BAR proteins could be found to be associated with newly formed macropinosomes. Cells were pulsed with dextran for 5 minutes, and the association of SNX1, SNX5, SNX9, SNX18 and SNX33 with macropinosomes was observed by high resolution confocal microscopy. SNX1 and SNX5 could be observed colocalizing with each other on discrete patches of the newly formed macropinosome (Figure 4A), and both SNX9 (Figure 4B) and SNX18 (Figure 4C) could be found on these early stage macropinosomes as well. SNX33 however, could not be observed being recruited onto any membrane within the cell, remaining cytosolic in distribution (Figure 4D). These results further support the notion that SNX1, SNX5, SNX9, and SNX18 regulate the formation of macropinosomes early in their lifecycle, as they can be found on the macropinosome body shortly after formation.

**Figure 5:**
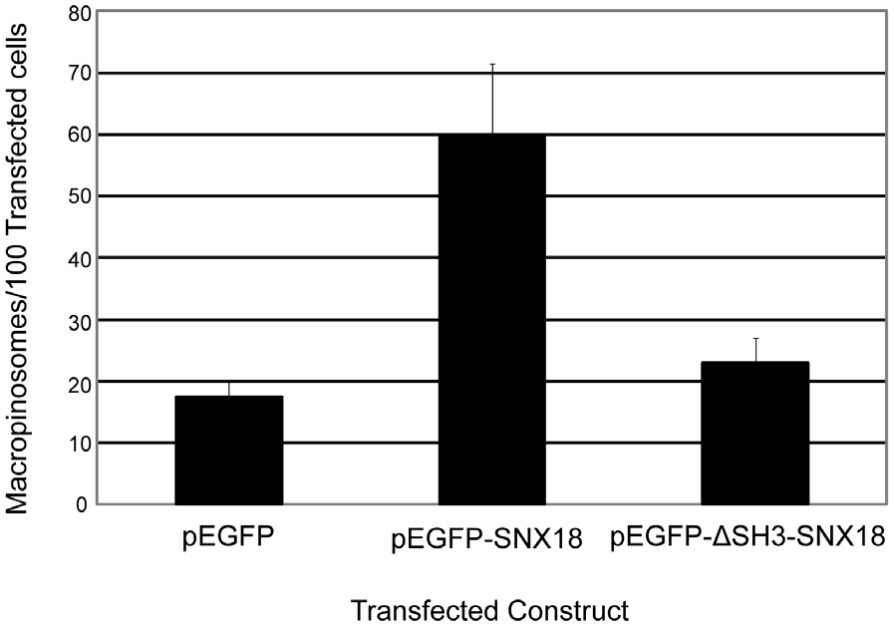
The SH3 domain of SNX18 is required for elevation of macropinosome formation. HEK-Flp-In cells transiently overexpressing pEGFP, pEGFP-SNX18, or pEGFP-ΔSH3-SNX18 were assayed for macropinosome formation as described in Materials and Methods. The mean number of macropinosomes/100 transfected cells was quantitated over 3 replicates of 500 transfected cells for each condition. Error bars denote S.E.M.

Following on from the observation that all the members of the SH3-PX-BAR subgroup – SNX9, SNX18, and SNX33 - upregulated macropinosome formation when overexpressed, we decided to specifically investigate the contribution of the SH3 domain within these proteins towards elevating macropinosome formation. Detailed functional protein domain analyses have previously been conducted for both SNX9 [41] and SNX33 [54], so we decided to focus on SNX18. We generated a pEGFP-ΔSH3-SNX18 construct where the first 60 residues corresponding to the SH3 domain have been deleted. This construct was then transfected into HEK-Flp-In cells, and assayed for its impact on macropinosome formation. It was observed that unlike the overexpression of pEGFP-SNX18, the elevation of pEGFP-ΔSH3-SNX18 did not result in a statistically significant increase in macropinosome numbers compared to cells expressing pEGFP (Figure 5). This suggests that the SH3 domain of SNX18 is required for SNX18-induced upregulation of macropinosome formation.

### Modulating PI(3,4,5)P_3_ levels affects macropinosome formation

The PX-BAR modules of SNX9 and SNX18 have specifically been reported to bind PI(4,5)P_2_ [46], a phosphoinositide which transiently increases in concentration on membrane ruffles before dropping in accordance with the subsequent enrichment of PI(3,4,5)P_3_ on the cell surface and the macropinocytic cup [30], [31], [32]. Given the necessity for PI(3)K activity in macropinosome formation [10], [28], the spatiotemporal distribution of these phosphoinositides early in the macropinocytic lifecycle, and the PI(4,5)P_2_ binding specificity of SNX9 and SNX18, we hypothesized that the regulation of phosphoinositide levels may play a significant role in the mechanism by which SNX9 and SNX18 upregulates macropinocytosis.

**Figure 6:**
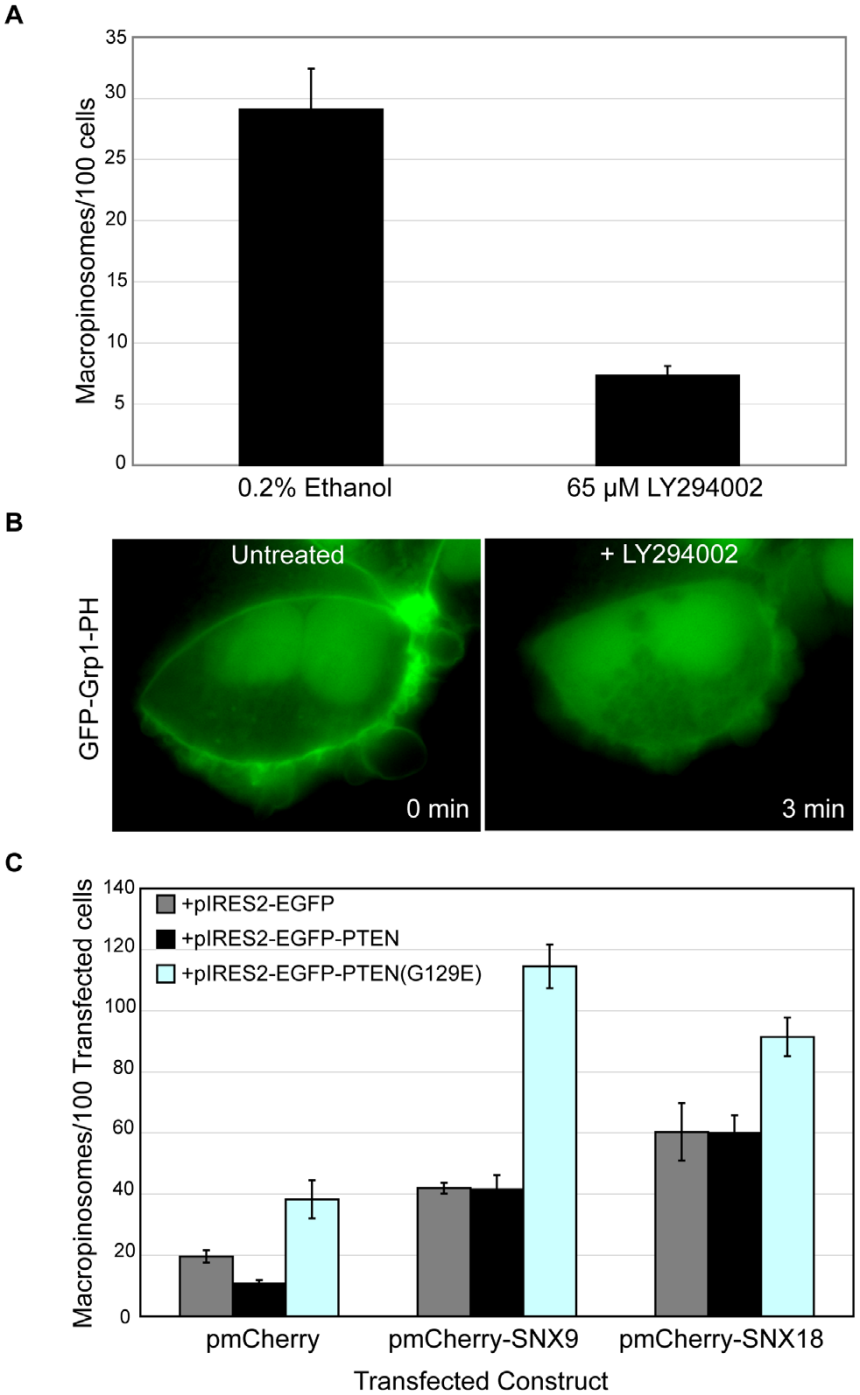
The modulation of PI(3,4,5)P_3_ levels affects macropinosome formation. A: HEK-Flp-In cell monolayers were treated with either 65 µM LY294002 or carrier (0.2% Ethanol) for 30 minutes at 37°C prior to pulsing with dextran-TR as described in Materials and Methods in the continued presence of the drug or carrier. The mean number of macropinosomes/100 cells was quantitated as described in Materials and Methods. Error bars represent S.E.M. B: HEK-Flp-In cell monolayers transfected with pEGFP-Grp1-PH for 24 hours were imaged using the 100× oil immersion objective of an Olympus IX-81 OBS Real Time microscope before and after treatment with 65 µM LY294002. Time following LY294002 treatment is indicated in bottom right hand corner of each panel. C: HEK-Flp-In cells transiently transfected with pIRES2-EGFP, pIRES2-EGFP-PTEN, or pIRES-EGFP-PTEN(G129E) were co-transfected with pmCherry, pmCherry-SNX9, or pmCherry-SNX18 and assayed for macropinosome formation as described in Materials and Methods. The mean number of macropinosomes/100 transfected cells was quantitated over 3 replicates of 500 transfected cells for each condition. Error bars denote S.E.M.

We investigated the validity of this hypothesis by first pre-treating cells with 65 µM LY294002 for 30 minutes to inhibit PI(3)K activity, and assessing the effect of this treatment on macropinosome formation relative to the carrier (0.2% ethanol). A 4-fold decrease in macropinosome formation (p<0.05) was observed in LY294002-treated cells as compared to carrier-treated samples (Figure 6A), consistent with previous observations [55]. We then examined the effect of inhibiting PI(3)K activity on the subcellular distribution of PI(3,4,5)P_3_ by transiently expressing a GFP fusion protein of the Grp1 PH domain (GFP-Grp1-PH), which has been reported to specifically bind PI(3,4,5)P_3_ [56]. GFP-Grp1-PH was observed on the plasma membrane in untreated cells (Figure 6B), consistent with previous reports that PI(3,4,5)P_3_ is localized on the cell surface [57]. Within 3 minutes of treatment with LY294002, the cell-surface labeling of GFP-Grp1-PH was ablated, and its distribution throughout the cell became predominantly cytosolic (Figure 6B). Together these results suggest the possibility that PI(3)K activity is required to maintain the levels of PI(3,4,5)P_3_ on the cell surface in order for macropinosome formation to occur.

We further investigated the role of PI(3,4,5)P_3_ levels in macropinosome formation by using the PI(3,4,5)P_3_ phosphatase PTEN to modulate intracellular PI(3,4,5)P_3_ [58]. Compared to cells transfected with the empty pIRES2-EGFP and pmCherry vectors, cells co-expressing pmCherry and pIRES2- EGFP -PTEN exhibited a 2-fold decrease in the number of macropinosomes formed (p<0.05) using the screening assay (Figure 6C). This suggests that by overexpressing pIRES2-EGFP-PTEN and thus promoting the dephosphorylation of the existing intracellular pool of PI(3,4,5)P_3_ to PI(4,5)P_2_, macropinosome formation was significantly attenuated. Moreover when the phosphatase-deficient mutant of PTEN, pIRES2-EGFP-PTEN(G129E) was co-expressed with the empty pmCherry vector, a 2 fold elevation in macropinocytosis was observed relative to control cells (p<0.05). PTEN(G129E) still possesses the capacity to bind PI(3,4,5)P_3_ but is unable to catalyse its dephosphorylation [59]. Elevated levels of PTEN(G129E) may competitively bind to, but not dephosphorylate PI(3,4,5)P_3_ thus lowering the rate at which PI(3,4,5)P_3_ is metabolized within the cell by endogenous PTEN and transiently elevating intracellular PI(3,4,5)P_3_. Intriguingly, cells coexpressing pIRES2-EGFP-PTEN(G129E) and pmCherry-SNX9 further exaggerated the already elevated levels of macropinosome formation in pmCherry-SNX9 and pIRES2-EGFP-coexpressing cells by 2.7 fold (p<0.05) (Figure 6C).The same was observed for cells coexpressing pmCherry-SNX18 and pIRES2-EGFP-PTEN(G129E), elevating macropinosome formation by 1.5 fold over pmCherry-SNX18 and pIRES2-EGFP coexpressing cells (p<0.05) (Figure 6C). Interestingly coexpression of pIRES2-EGFP-PTEN with either pmCherry-SNX9 or pmCherry-SNX18 did not significantly alter the frequency of macropinosome formation as compared to cells coexpressing pIRES2-EGFP with pmCherry-SNX9 or pm-Cherry-SNX18 respectively (Figure 6C); this is possibly because elevating the levels of SNX9 or SNX18 is able to overcome the lowering of intracellular PI(3,4,5)P_3_ in regulating macropinosome formation. These data suggests that the actions of PTEN(G129E) in potentially elevating intracellular PI(3,4,5)P_3_ are synergistic with SNX9 and SNX18 in elevating macropinosome formation, implicating the role of PI(3,4,5)P_3_ in SNX9 and/or SNX18-mediated regulation of macropinosome formation.

## Discussion

In this study, we utilized image-based quantitative analyses to implement a gain-of-function screening assay for assessing macropinosome formation. The sensitivity of the assay was validated by detecting an acute 2 fold elevation in macropinosome formation in response to EGF treatment, and a 4.54 fold reduction in macropinosome numbers upon amiloride treatment. The assay was also tested against the overexpression of known regulators of macropinocytosis; transient overexpression of SNX5 significantly increased macropinosome formation, as did Rab5 and Rabankyrin-5 overexpression. Moreover given that Rabankyrin-5 is a Rab5 effector, the significantly higher levels of macropinocytosis in Rabankyrin-5 overexpressing cells relative to those overexpressing Rab5 demonstrates the sensitivity of the screening assay in detecting the quantitative difference between modulating upstream and downstream components of the macropinocytic pathway.

The capacity of the assay to specifically quantify macropinocytosis and not other endocytic pathways was also validated. The main distinguishing factors of macropinosomes are their large size relative to other endocytic organelles (0.2–5 µm as compared to up to 200 nm in diameter) and their derivation from the plasma membrane. Overexpression of the Rab5Q79L mutant is known to promote homotypic endosomal fusion [53], [60], resulting in the formation of large endocytic structures that fulfill the size criterion of macropinosomes. Only dextran-positive structures formed within 5 minutes of dextran uptake were counted in the assay however, and the Rab5-Q79L-positive enlarged endosomes were not found to associate with any dextran-positive macropinosomes. Moreover Rab5-Q79L overexpression had no detectable impact on macropinosome formation. These data provide assurance that the dextran-positive structures quantified in the screening assay are derived from the cell surface and not a result of the homotypic fusion of internal endosomal membranes, further validating its specificity in measuring macropinocytosis.

Following their formation at the cell surface, macropinosomes undergo a series of maturation events before either recycling back to the cell surface [61], or fusing with the late endosomes/lysosomes [62]. The intracellular fate of macropinosomes is largely dependent on cell-type; in A431 cells, macropinosomes recycle back to the plasma membrane [61] whereas within macrophages, macropinosomes progressively acquire molecules conferring early and late endosomal identity, finally being delivered to the late endosome/lysosome [62]. HEK-Flp-In cells were used as the experimental system within this study and have been previously observed to possess a macropinocytic lifecycle similar to that of macrophages [63], with very infrequent macropinosome recycling events as monitored by time-lapse video microscopy. It is therefore unlikely that macropinosome recycling back to the cell surface within our experimental system would confound the measurement of macropinosome formation.

Additionally, the screening assay defines newly formed macropinosomes as endocytic organelles >0.5 µm in diameter formed within a 5 minute window of dextran internalisation. These size and temporal criteria minimize the likelihood that downstream maturation events within the macropinocytic lifecycle may confound the measurement of macropinosome formation. As evidenced by the inability of Rab5-Q79L overexpression to influence macropinosome formation, the contribution of homotypic fusion to macropinosome numbers measured by the screening assay is minimal. Furthermore although the extensive membrane tubulation driven by elevated levels of SNX-PX-BAR proteins reduces the surface area and volume of macropinosomes 3–8 minutes post formation [14], the size of these organelles still significantly exceeds 0.5 µm in diameter by the end of this timeframe [64]. Hence within the 5 minutes of dextran uptake measured in the screening assay, it is unlikely that any membrane tubulation will significantly alter the size of the macropinosomes to be excluded by the image analysis methodology. Finally the observation of macropinocytic events within 5 minutes of dextran internalisation should not be affected by macropinosome degradation through fusion with the late endosomes/lysosomes, as this late maturation event occurs 20–25 minutes post formation [63]. Thus, it is likely that the screening assay is able to specifically quantify the number of newly formed macropinosomes without being confounded by early or late maturation events.

The application of this gain-of-function screening assay to the SNX-PX-BAR family found that SNX1, SNX5, SNX9, SNX18 and SNX33 all elevated macropinosome formation when overexpressed. SNX1 has been observed to interact and form heterodimers with SNX5 by several groups [14], [65] in spite of limited evidence to the contrary [66]. Like SNX5, SNX1 overexpression also changes the frequency of macropinosome formation, and together with their colocalization on newly formed macropinosomes early in the formation process suggest that the two proteins are acting in complex as part of a common mechanism in macropinocytosis.

It is intriguing that apart from SNX1 and SNX5, the remaining positive candidates from the gain-of-function screen - SNX9, SNX18 and SNX33 - all belong to the SH3 subgroup of the SNX-PX-BAR family. All three SH3 subgroup proteins possess an N-terminal SH3 domain, which has been shown to be necessary for interacting with a wide variety of endocytic and actin-regulatory molecules [46], [67]. SNX9 possesses arguably the strongest link to macropinocytosis, as it has been mechanistically linked to macropinosome formation by virtue of its role in actin assembly. It has been demonstrated to drive N-WASP activation at the cell surface, interact with the Arp2/3 complex, and consequently promote actin polymerization [40], [68]. Moreover, siRNA-mediated depletion of SNX9 results in a downregulation of fluid-phase uptake [40].

SNX33 has been shown to interact with SNX9 [54], so it is likely that they share a similar mechanism of action in elevating macropinocytosis. SNX33 was also shown to directly interact with WASP and induce actin-polymerization in the perinuclear space [54]. However, we were unable to observe SNX33 recruitment on newly formed macropinosomes or any membrane throughout the cell, as its subcellular distribution is cytosolic. This may be due to high overexpression levels perturbing membrane recruitment of SNX33, as endogenous antibodies to SNX33 have revealed a punctate distribution throughout the cytoplasm [54]. This may also be cell-type specific, as a similar recruitment to puncta was observed by overexpressing an epitope-tagged SNX33 construct in HeLa cells [46]. As observed cytosolic SNX33 may still interact with WASP to drive actin polymerization in the pernuclear space, initiating membrane ruffling and macropinocytosis.

The implication of SNX18 in macropinosome formation further solidifies the involvement of the SNX-PX-BAR family in macropinosome formation, and SNX18 can also be found on early-stage macropinosomes. This result corroborates recent findings that place SNX18 on circular dorsal ruffles and membrane ruffles in regulating clathrin-independent fluid-phase endocytosis [67]. The observation that the protein-scaffolding SH3 domain of SNX18 is required for an elevation in macropinosome formation may suggest that an intact association between SNX18 and other key molecular effectors is required in driving macropinocytic uptake. Indeed, SNX18 has been found in complex with SNX9, N-WASP and dynamin at the cell surface [46], [67] and it is possible that SNX9 and SNX18 regulate macropinocytosis through a common mechanism. This is likely to be through the regulator of actin polymerization N-WASP, with which they both can associate [40], [67], [68]. Strategic siRNA-mediated depletion of each of these molecules in turn would shed more light on the dynamics of their contribution towards macropinocytosis.

Given the requirement for PI(3)K activity in macropinocytosis and the propensity for the PX domain to target proteins to phosphoinositide-rich membranes [35], a reasonable assumption would be that the contribution of phosphoinositides is important in the upregulation of macropinosome formation as mediated by SNX-PX-BAR proteins. Interestingly, modulating the levels of PI(3,4,5)P_3_ by overexpressing PTEN or its phosphatase-deficient mutant PTEN(G129E) revealed a correlation between PI(3,4,5)P_3_ levels and macropinosome formation. SNX9 and SNX18 have both been reported to preferentially bind to PI(4,5)P_2_ [46], potentially indirectly facilitating the conversion into PI(3,4,5)P_3_ by interacting with class I PI(3)K to elevate macropinosome formation. Our findings that coexpressing PTEN(G129E) and SNX9 or SNX18 was synergistic in the elevation of macropinosome formation support this model, although further work is needed in characterizing potential interactions between PI(3)K and SNX9 and/or SNX18. It is unlikely that SNX1 and SNX5 regulate PI(3,4,5)P_3_ formation through this mechanism, as SNX1 binds to PI(3)P and PI(3,5)P_2_ [44], while SNX5 binds to PI(3)P and PI(3,4)P_2_ [45]. A transition from PI(3,4,5)P_3_ to PI(3)P has been observed on the macropinosome shortly post formation however [31], [32], and SNX1 and/or SNX5 may be temporally recruited to the macropinosome through the interaction between their PX domains and PI(3)P. The PI-binding specificity of SNX33 is still yet to be determined.

In conclusion, the characterization of macropinocytosis requires the detailed identification of specific molecular components that regulate different stages of its endocytic lifecycle. This study has contributed to the understanding of macropinocytosis by uncovering 5 members of the SNX-PX-BAR family in the regulation of macropinosome formation. By first understanding the molecular networks involved in the initiation of this endocytic pathway, further characterization of the downstream coordination required for subsequent stages in the macropinocytic lifecycle can be conducted within the appropriate molecular context.

## Materials and Methods

### Antibodies and DNA constructs

The goat-anti-mouse IgG-Cy3, dextran conjugated to tetramethylrhodmaine (dextran-TR) and Alexa647 (dextran-647) (10,000 MW) antibodies were purchased from Molecular Probes, Invitrogen (USA). The SNX1 antibody was purchased from BD transduction laboratories (Australia). The polyclonal SNX5 antibody was a kind gift from Jet Phey Lim (Bio21 Melbourne).

The construct pEGFP-C1 was obtained from Clontech. pEGFP-Rab5 [69], pEGFP-Rab5Q79L [70], and pEYFP-Rabankyrin 5 [34], are as described previously. pIRES2-EGFP-PTEN and pIRES2-EGFP-PTEN(G129E) were generated by subcloning the open-reading frames of PTEN and PTEN(G129E) [59] into pIRES2-EGFP using BamHI and EcoRI. pEGFP-SNX1, pEGFP-SNX2, pEGFP-SNX4, pEGFP-SNX5, pEGFP-SNX6, pEGFP-SNX7, pEGFP-SNX8, pEGFP-SNX9, pEGFP-SNX18, pEGFP-SNX30, pEGFP-SNX32, and pEGFP-SNX33 were generated by amplifying their full length open reading frame by polymerase chain reaction (PCR) and the resulting PCR products cloned into pEGFP-C1. pmCherry-SNX18 was generated by amplifying the full length open reading frame of SNX18 and cloning the resulting PCR product into pmCherry-C1. pEGFP-ΔSH3-SNX18 was generated by amplifying residues 61-615 of SNX18 and cloning the resulting PCR product into pEGFP-C1. We thank Dr Heung-Chin Cheng (The University of Melbourne) for his generosity in supplying the original PTEN plasmids.

### Cell culture and transfection

HEK-Flp-In cells (Invitrogen) were maintained in DMEM supplemented with 10% (v/v) FCS and 2 mM L-glutamine (Invitrogen) in a humidified air/atmosphere (5% CO2) at 37°C. Cells were seeded onto glass coverslips pre-treated with poly-L-lysine (Sigma, USA), and grown to 90% confluence over 3 days prior to transfecting with Lipofectamine 2000 as per manufacturer's instructions (Invitrogen). 0.8 µg of DNA was used along with 2 µl of Lipofectamine 2000 per well in a 24 well plate.

### EGF treatment and pharmacological agents

EGF and Amiloride were obtained from Sigma (USA). LY294002 was purchased from Merck (Australia). YM201636 was purchased from Symansis: Cell Signalling Science.

For EGF treatment, cells were serum starved for 16 hours before being treated in the presence or absence of 100 ng/mL EGF for various timepoints. Amiloride (1 mM) and LY294002 (65 µM) were each applied to cells at 37°C for 30 minutes. All experiments following this incubation period were done in the continued presence of the compound(s).

### Streptolysin O permeabilisation

Cell monolayers were transferred to 4°C and washed three times with 0.45 mM CaCl_2_ 1 mM MgCl_2_ PBS. The cells were then treated with 80 U/mL Streptolysin O (Sigma, USA) for 5 minutes at 4°C to only permeabilize the plasma membrane before washing in 0.45 mM CaCl_2_ 1 mM MgCl_2_ PBS and incubating with 37°C PBS for 5 minutes prior to fixation in 4% PFA.

### Indirect Immunofluorescence

Cell monolayers were fixed in 4% PFA at 4°C for 30 minutes, and washed in PBS three times prior to permeabilisation with 0.1% TritonX100 for 10 minutes. Samples were washed in 2% BSA blocking solution three times, and incubated with the primary antibody for 1.5 hours at room temperature. The primary antibody was removed and samples washed again in blocking solution three times before incubating with appropriate secondary antibodies conjugated to specific fluorophores for 1 hour at room temperature. Samples were then washed with blocking solution and mounted onto microscope slides for imaging.

### Live Cell Imaging

HEK-Flp-In cell monolayers were seeded onto 35 mm glass-bottom dishes (MatTek Corporation) coated with poly-L-lysine (Sigma-Aldrich). 24 hours post transfection, the samples placed in 10% FCS CO2-independent media (Invitrogen) and maintained at 37°C during live-cell imaging. Using an Olympus IX-81 OBS Real Time microscope, samples were illuminated with a Xenon lamp to sequentially capture GFP epifluorescent images on the 100× oil immersion objective.

### Macropinosome Formation Screening Assay

HEK-Flp-In cell monolayers were incubated with 100 µg/mL dextran for 5 minutes at 37°C, before being washed twice with 4°C PBS and fixed in 4°C 4% PFA for 30 minutes. Samples were then imaged on an LSM 510 Meta confocal scanning microscope on the 40× objective, capturing 3×5 µm Z stacks. Macropinosomes were quantitated by collapsing the Z-stacks of the images captured using the “Average Intensity” function in ImageJ 1.42q (NIH). The original RGB image is converted to 8 bit grayscale format, and the “Subtract Background” functionality employed with input variable of 8.0 pixels. Macropinosomes were then selected based on fluorescent intensity (>100) and size (>0.5 µm in diameter). To specifically determine the number of macropinosomes within transfected cells, areas of the image corresponding to macropinosomes are used to create a mask which is then super-imposed upon the GFP channel and the GFP fluorescent intensity measured. GFP intensity higher than the background signal (>20) indicates a macropinosome within a transfected cell. The number of cells per field of view was quantitated by the number of DAPI-positive nuclei or GFP-positive cells.
